# Metal Oxide Semi-Conductor Gas Sensors in Environmental Monitoring

**DOI:** 10.3390/s100605469

**Published:** 2010-06-01

**Authors:** George F. Fine, Leon M. Cavanagh, Ayo Afonja, Russell Binions

**Affiliations:** Department of Chemistry, University College London, 20 Gordon Street, London WC1H 0AJ, UK; E-Mails: georgefine@gmail.com (G.F.F.); l.cavanagh@ucl.ac.uk (L.M.C.); ayo.afonja@ucl.ac.uk (A.A.)

**Keywords:** metal oxides, semiconductor, zeolites, environmental monitoring

## Abstract

Metal oxide semiconductor gas sensors are utilised in a variety of different roles and industries. They are relatively inexpensive compared to other sensing technologies, robust, lightweight, long lasting and benefit from high material sensitivity and quick response times. They have been used extensively to measure and monitor trace amounts of environmentally important gases such as carbon monoxide and nitrogen dioxide. In this review the nature of the gas response and how it is fundamentally linked to surface structure is explored. Synthetic routes to metal oxide semiconductor gas sensors are also discussed and related to their affect on surface structure. An overview of important contributions and recent advances are discussed for the use of metal oxide semiconductor sensors for the detection of a variety of gases—CO, NO_x_, NH_3_ and the particularly challenging case of CO_2_. Finally a description of recent advances in work completed at University College London is presented including the use of selective zeolites layers, new perovskite type materials and an innovative chemical vapour deposition approach to film deposition.

## 1. Introduction

Since 1962 it has been known that absorption or desorption of a gas on the surface of a metal oxide changes the conductivity of the material, this phenomenon being first demonstrated using zinc oxide thin film layers [1]. The sensitivity of a surface to a gas can be as low as parts per billion (ppb) [2–5]. It is highly desirable that metal oxide semiconductor sensors have a large surface area, so as to adsorb as much of the target analyte as possible on the surface, giving a stronger and more measurable response (especially at low concentrations). Advances in fabrication methods have enabled the production of low-cost sensors with improved sensitivity and reliability compared to those formed using previous methods [6]. Production costs are kept low due to the simplicity of metal oxide semiconductor sensor devices. Their ability to be produced quickly and on a large scale with easily controllable processes makes them a desirable technology to exploit. This paper aims to: (i) introduce the fundamental reasons for sensing gases, (ii) discuss sensor response mechanisms in metal oxide semiconductor sensors, and (iii) show how non-target gases can interfere with the response of such a sensor. The review will then present a summary of recent advances on sensors that have been developed for specific gases such as carbon dioxide and carbon monoxide, and in the final section discuss new approaches developed in our labs at University College London such as the ways in which zeolites can be used to increase specifity, selectivity and efficiency of sensors.

### 1.1. Basic Theory

#### 1.1.1. Band Theory

**Figure 1.**
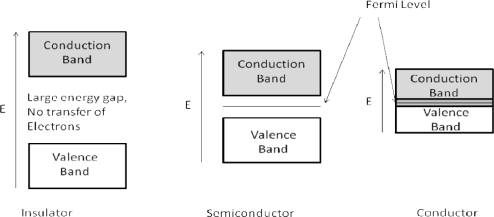
Schematic band diagrams of an insulator, semi-conductor and conductor—Note the small gap in the semiconductor, where electrons with sufficient energy can cross and the overlapping of the bands in the conductor.

Band theory states that within a lattice there exists a valence band and a conduction band. The separation between these two bands is a function of energy, particularly the Fermi level, defined as the highest available electron energy levels at a temperature [7]. There are three main classes of material in band theory (see Figure 1).

Insulators have a large gap between the valence and conduction band (typically taken to be 10 eV or more), as such a lot of energy is required to promote the electron in to the conduction band and so electronic conduction does not occur. The Fermi level is the highest occupied state at T = 0 [8]. Semiconductors have a sufficiently large energy gap (in the region of 0.5–5.0 eV) so that at energies below the Fermi level, conduction is not observed. Above the Fermi level, electrons can begin to occupy the conduction band, resulting in an increase in conductivity. Conductors have the Fermi level lying within the conduction band.

#### 1.1.2. Band Theory Applied to Sensors

Band theory as applied to gas sensors has been the subject of intense study for a number of years [9–11]. The target gas interacts with the surface of the metal oxide film (generally through surface adsorbed oxygen ions), which results in a change in charge carrier concentration of the material. This change in charge carrier concentration serves to alter the conductivity (or resistivity,) of the material. An n-type semiconductor is one where the majority charge carriers are electrons, and upon interaction with a reducing gas an increase in conductivity occurs. Conversely, an oxidising gas serves to deplete the sensing layer of charge carrying electrons, resulting in a decrease in conductivity. A p-type semiconductor is a material that conducts with positive holes being the majority charge carriers; hence, the opposite effects are observed with the material and showing an increase in conductivity in the presence of an oxidising gas (where the gas has increased the number of positive holes). A resistance increase with a reducing gas is observed, where the negative charge introduced in to the material reduces the positive (hole) charge carrier concentration. A summary of the response is provided in Table 1.

### 1.2. A Model for Gas Interaction

#### 1.2.1. A p-Type Sensor Response

A simple model for the response of a p-type sensor is demonstrated by Equations 1 and 2; showing the adsorption of an oxygen atom to the surface of the material, causing ionisation of the atom and yielding a positive hole (p^+^), demonstrated by (1). The positive hole and the ion can then react with a reducing gas such as carbon monoxide, forming carbon dioxide (k_2_) or be removed through interaction with each other (k_–1_) [12] (2) The difference in charge carrier concentration (in this case the positive hole) is manifest in a resistance change between the sensor’s electrodes and read by the measurement circuitry:

(1) $$1/2\;O_{2}\overset{k_{1}}{\underset{k_{-1}}{⇌}}O^{-}+p^{+}$$

(2) $$p^{+}+O^{-}+\text{CO}\overset{k_{2}}{\to }{\text{CO}}_{2}$$

#### 1.2.2. The Equivalent Circuit Model

The response model, as described by Naisbitt [13] *et al.*, which includes the influence of sensor microstructure is a refinement of the conventional response model that is found to apply of over a limited range of examples only, and assumes that the absorbed species on the surface of the metal oxide is the O^2−^ species; a species that is unlikely to be included as it is energetically unfavourable (Equation 3). Describes a relationship where the change in resistance is proportional to the concentration of the gas (in this example carbon monoxide, CO) and a sensitivity parameter *A* (the sensitivity parameter is constant for a given material at a given temperature) where R is the resistance after exposure to analyte gas and R_0_ the baseline resistance (Equation 4):

(3) $$1/2\;O_{2}\overset{k_{4}}{\underset{k_{-4}}{⇌}}O^{2-}+2P^{+}$$

(4) $$R/R_{0}=1+A\left[\text{CO}\right]$$

Instead, Naisbitt *et al.* propose that Equation (1) is more likely, but to account for non-linear responses, there must be other factors influencing the response. First, the assumption is made that the only parts of the material that exhibit a response to the target gas, are the areas where the gas can reside and interact at the surface.

**Figure 2.**
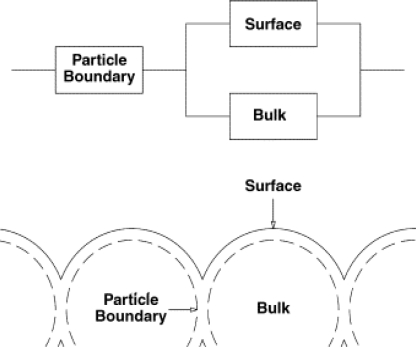
Demonstrating the structure of the materials, and the positions of the surface, bulk and particle boundary. Figure adapted with permission from Naisbitt *et al*. [13]. The model assumes the gas sensitivities of the surface and particle boundary are the same.

Thus, the material is split into three regions (Figure 2): (i) the surface, (ii) the bulk (inaccessible to the target gas), and (iii) the neck or particle boundary (below this boundary, the material is no longer defined as a surface). The distance between the surface and the particle boundary is called the Debye length; the distance at which charge separation can occur. The Equation for the response in this model is found to be Equation (5):

(5) $$G_{T}=\gamma \text{PB}(1+A[\text{CO}])+1/[(1/\gamma_{B})+(1/\gamma S(1+A[\text{CO}])$$

where G_T_ is the response (G_T_= R_T_/R_T,0_), R_T_ is the total sensor resistance R_T,0_ is the baseline resistance in clean, dry air and each γx = R_x,0_/R_T,0_ gives (x denotes particle boundary, PB; bulk, B; or surface, S. So, γ is the ratio of the baseline of x to the total baseline of sensor resistance [7].

This work shows that the response time is directly related to the grain size and the size of the particle boundary in the material. The response model is different for n-type and p-type semiconductors. In forming the baseline resistance, oxygen is adsorbed on to the surface and abstracts electrons from the material; hence, this process will determine R_0_. The resistivity of the p-type decreases relative to the bulk, and will increase for the n-type. The relative contributions from the three resistors in the model then differ. For very small grain sizes, the grain can be considered to contain no bulk area at all (so the whole grain is considered to contribute to the surface area) in this instance, the simpler model and Equation (4) is an adequate model for the response. If one considers the other extreme, where the grains are so large the contribution to the resistance or conductivity is negligible, the surface can be deemed to have a constant resistance. This model is expected to be generally applicable to both p and n-type sensors.

### 1.3. Sensor Response Disruption

The presence of other gases in the sensor headspace is important. The measurement of conductivity should ideally be only for the target analyte. If a contribution to the charge carrier concentration (whether this be an increase or decrease) comes from another gas, the sensor reading will be inaccurate, providing a false measurement. Some of the important disrupting gases are listed below.

#### 1.3.1. Ozone

Oliver *et al.* showed that when the concentration of oxygen vacancies is high, the concentration of electrons is high on the surface [14] (5). This would elevate conductivity (for n-type material), but upon the introduction of ozone on the surface it is reduced, filling the vacancies (considered as negative holes) and lowering the conductivity (6). (Equations 6 and 7 in Kröger-Vink notation) [6].

(6) $$W_{w}^{\prime }⇄W_{w}^{x}+e^{-}$$

(7) $$O_{3}+2W_{w}^{\prime }+V\ddot{o}\to {2W}_{w}^{\prime }+O_{o}^{x}+O_{2}$$

If ozone is present in significant concentrations, the response of the material will be altered, leading to misleading figures as to the presence or concentration of the target gas.

#### 1.3.2. Water

Water is thought to interact with the metal oxide, altering the conductivity of the film [15]. Water molecules form hydroxyl (OH^−^) ions on the surface, directly introducing electrons that increase the conductivity of an n-type sensor (having little affect on a p-type sensor). The remaining hydrogen atoms react with the surface oxygen atoms, forming negative holes and increasing conductivity. Work by Korotcenkov *et al.* [16] investigated the effect of humidity on the response of a SnO_2_ film, with the film demonstrating a decreased response time and larger responses in the presence of water. The authors suggest that the effect of water should be taken in to account when detecting reducing gases.

#### 1.3.3. Volatile Organic Compounds

Research has demonstrated the indiscriminate response of SnO_2_ films to varying hydrocarbons. Wang *et al.* [17] demonstrated that a metal oxide semiconductor sensor responds to methyl, ethyl, isopropyl and butyl alcohol. Indium tin oxide has been shown to respond to methanol, ethanol, butanol, and acetone by Vaishanv *et al.* [18]. Selectivity and control of sensor response is a major challenge in gas sensing using metal oxide semiconductor devices; trace levels of volatile organic compounds are present in virtually all environments.

### 1.4. Factors Influencing Sensor Design

Many factors must be addressed when designing a new metal oxide semiconductor gas sensor; such as the material’s sensitivity and specificity to the gas in question or if the sensitivity of the material is appropriate for the application. The same gas sensor may not be appropriate in two different environments: a carbon dioxide sensor for the inside of a car exhaust should be designed for high concentrations, whereas one car cabin air quality should be far more sensitive to carbon dioxide at lower concentrations. The sensor would not be accurate enough to register concentration changes of 10 ppm if its sensitive range is 1,000–10,000 ppm. The surrounding pollutant gases will also affect the sensor gas response. As mentioned above (Section 1.3), certain gases will change the charge carrier concentration despite them not being the target analyte. A sensor in a photocopying room for example, might show unexpected results because the concentration of ozone is elevated; the ozone gas may interact with the film, nullifying or increasing a response. In this case, selecting a material that does not respond to ozone, altering the surface of the film or employing a suitable external filter to inhibit ozone interacting with the surface is necessary.

### 1.5. Techniques for Sensor Fabrication

Many fabrication methods have been used in the production of metal oxide semiconductor sensors. Factors that must be considered when selecting the production technique include; expense (if the films are expensive, the demand will be low and will have only limited applications), purity, porosity (if the material is highly porous, the surface area available to the gas for interaction will be far higher, giving a higher sensitivity), reliability and reproducibility [1]. Common techniques for making the metal oxide films for gas sensors are mainly chemical vapour deposition (CVD), screen-printing of ceramic powders, sol-gel techniques and physical vapour deposition (PVD).

#### 1.5.1. Screen Printing

**Figure 3.**
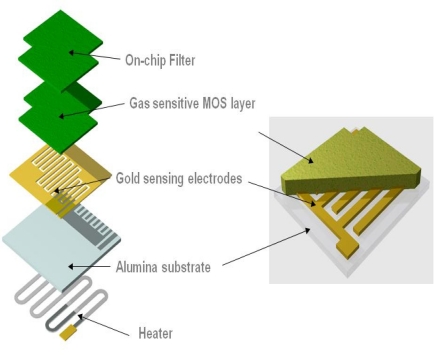
Demonstrating the production of a gas sensitive film on a sensor substrate (diagram courtesy of Capteur Sensors and Analysers).

Screen-printing is widely used in industry and the most widely used method for producing metal oxide semiconductor gas sensors commercially [8,18]. Screen-printing involves pushing an ink through a porous layer or mesh, which is suitably masked to produce the required layout on the substrate. The ink essentially contains the material to be deposited, dispersed in a viscous vehicle and is printed on of the substrate. Once the ink is has been deposited, the print can be heated to remove the vehicle, leaving a solid material on the specific target area. Figure 3 indicates the general form of commercial metal oxide semiconductor gas sensors.

#### 1.5.2. Chemical Vapour Deposition (CVD)

**Figure 4.**
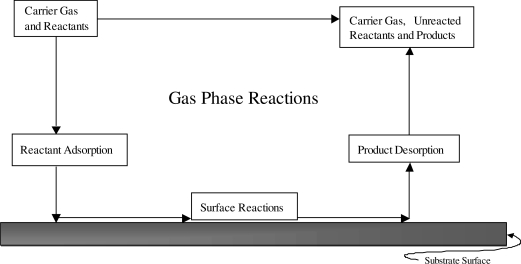
A basic outline of the CVD method, depositing a film on to the substrate surface. Figure adapted with permission from [22].

In CVD [19], a heated substrate is exposed to a precursor or controlled mixture of precursors in the vapour phase. The vapour(s) then react or decompose on the heated substrate forming a film of the desired material (Figure 4). Examples of different CVD techniques include atmospheric pressure CVD (APCVD), aerosol assisted CVD (AACVD); where non-volatile precursors have to be made in to an aerosol before deposition [20,21], or rapid thermal CVD where the substrate is heated, not the surroundings, avoiding unwanted reactions when the precursors are in the air. CVD gives control over important aspects of gas sensing materials; properties such as porosity, grain size and thickness can all be well controlled using CVD [3]. The speed of film growth is an advantage of the technique, with a rate of up to 1 μm a minute for processes that occur at atmospheric pressure. The problem of reactions in the gas phase in CVD can be addressed by reducing precursor concentration, though in doing so, the film growth rate is also reduced. Suitable precursors can also be an issue in CVD. Precursors must be volatile enough in order to allow successful delivery to the heated substrate, but gas phase reaction and decomposition must be minimal, while having a structure that is thermodynamically able to decompose on the substrate giving the desired product is an area of significant research in producing any viable film.

APCVD has been used to deposit films of tungsten oxide for use as gas sensors by Binions *et al.* [23]. The gas sensors were shown to give a good response in the presence of ethanol. Williams *et al*. [24,25] have used CVD methodologies to produce films of WO_3_ and chromium titanium oxide. This work demonstrated that the desirable high porosity of the gas sensitive film is obtainable via CVD routes. The suitability for the material as a gas sensor was not tested, but the author suggested the properties might be suitable for gas sensing.

Blackman *et al.* [26–28] have used AACVD to produce high surface area, nanostructured tungsten oxide films with various precursors, with their gas sensing properties investigated. Choy [29] observes that AACVD is a useful technique when forming oxides and the aerosol is readily surrounded by oxygen, for larger scale applications, it could be favoured as it is a relatively low-cost technique. The area of AACVD is promising, but in industry it is proving difficult to produce a repeatable and controlled aerosol. A solution has not been found to tackle this problem. While the advantageous capabilities that this process has in producing good films has been demonstrated in the lab, the process has yet to successfully adapted by industry.

#### 1.5.3. Spray Pyrolysis

**Figure 5.**
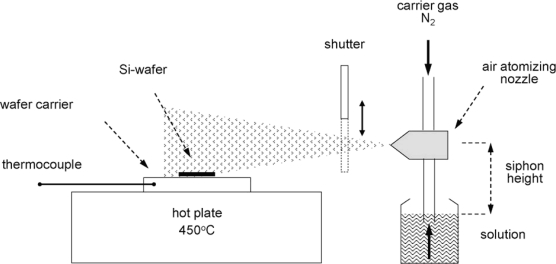
Demonstrating the spray pyrolysis technique for depositing thin films. Figure adapted with permission from Tischner *et al.* [30].

Spray pyrolysis (Figure 5) is a technique where the reactants for the film are sprayed on to the target substrate. The deposited droplets then react on the surface when the substrate is heated, forming the desired film [30].

#### 1.5.4. Sol-Gel

Sol-gel has been utilised for making gas sensitive films [14,31,32]. A recent report by Khun *et al.* uses the sol-gel method to synthesise SnO_2_ particles, which then demonstrated a response in the presence of Ammonia. The sol-gel process involves the formation of a solution (sol)—a colloidal suspension of solid particles. The sol can then undergo gelation (gel—where cross linking between particles occurs), this can give new materials with other properties. In the case of ceramic film formation, the sol then undergoes evaporation, giving a highly porous xerogel film. Upon heating, the film then forms a dense ceramic glass on the surface.

#### 1.5.5. Physical Vapour Deposition (PVD)

**Figure 6.**
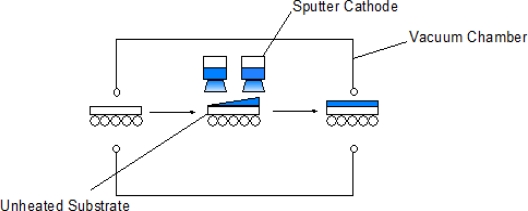
Schematic of the PVD process, adapted with permission from Kanu *et al.* [19].

In PVD techniques (Figure 6) the material to be deposited is put into the gas phase by either evaporation through heating or by sputtering (bombardment of the material by ions) [33,34]. A reactive gas is introduced, where the gas atoms react with the metal vapour, forming a compound, which is then deposited on the substrate, giving a process where there is control over the overall property of the coating. The resultant film is well bound to the substrate. Michel *et al.* used magnetron sputtering to produce SnO_2_ films [35]. The films were shown to have a level of conductivity, and upon exposure to hydrogen, the conductivity was shown to increase. Gupta *et al.* [36] produced a film of tin oxide by magnetron sputtering that demonstrated sensitivity to liquid petroleum gas. PVD is performed under vacuum; on large scale this can be an expensive technique. Growth rates, around 10 nm an hour makes this technique less suitable for high throughput for industrial applications.

#### 1.5.6. Drop Coating

Drop coating is a process by where a paste is made of the desired metal oxide powder and a suitable solvent; the paste is then deposited onto a substrate surface (in this case a gas sensor substrate) usually by controlled injection using a pipette. The deposited layer is subsequently fired to remove the solvent and attempt to improve adherence to the substrate [37]. The composition of the paste and nature of the dropping has a large influence on the film microstructure and subsequent sensor performance [38].

#### 1.5.7. Comparison of Synthetic Techniques

The advantages and disadvantages of each synthetic technique for the production of metal oxide semiconductor gas sensors will be discussed. Physical vapour deposition techniques require vacuum or reduced pressure conditions, which are time-consuming to achieve and maintain, and expensive evaporation/sputtering/ablation equipment, which increases production costs. Therefore, economic factors restrict the commercial applications of PVD. However, this does allow for the more efficient use of precursors and facilitates the production of ultra thin films, in comparison to CVD. PVD may operate at lower temperatures; hence, is compatible with a variety of substrates. Given that no chemical reaction takes place in PVD, as opposed to CVD, careful precursor selection is not a main concern. However, the purity of the target is required to deposit non-contaminated thin films. A variety of target can be incorporated into the system without difficulty, developing multilayer arrays.

Sol-gel methods are straightforward to operate, but the time required to establish the sol is important for obtaining the desired product, thus can be a slow multi-step process. Full coverage of the substrate with moderately even thickness can be achieved (which can be tricky to control over larger substrates), by using readily available precursors, although these are often expensive. Dopants may be easily introduced and the sol-gel process has low processing temperatures.

CVD is a non-line-of-sight process (unlike PVD) with high deposition rates at relatively low temperatures, thus depositing films with good conformal coverage and enables the synthesis of pure and uniform thin films, which generally exhibit good adhesion. CVD produces dense films which is a disadvantage for gas sensors as the contribution from the bulk resistance is substantially increased (Figure 2). However, a wide variety of growth morphologies are possible including nanowires and nanoparticles, which may lower this bulk contribution and increase surface area.

Binions *et al*. [39] compared CVD and screen-printing using gallium, antimony and tin oxides. It was found that sensors produced using CVD responded best to reducing gases at lower temperatures (450 °C), screen-printed films showed a larger response at higher temperatures (500 °C).

Drop coating has been shown to be a highly versatile process that is simple to perform that can widely influence sensor properties [38]; as such it finds use in some commercial devices. Screen-printing is also an industry standard technique. This is not surprising as it is found that the production of thick films leads to much more reliable sensor devices than thin film types [39]. Thin film devices are often more sensitive, although not necessarily more selective, but suffer from high baseline resistance due to a lower overall number of charge carriers whether in the bulk or on the surface of the material.

## 2. Carbon Monoxide Sensors

### 2.1. Reasons for Detecting Carbon Monoxide

Carbon monoxide (CO) is a colourless gas, with no odour, making it undetectable to humans. It is the leading cause of poisoning in the United States and may account for more than 50% of fatal poisonings reported in many industrial countries (Table 2) [40–43]. The gas has been shown to bind irreversibly to the iron centre of haemoglobin, the oxygen transport molecule in blood. The irreversible binding means that oxygen can no longer be absorbed, and at high levels of exposure this results in death. The maximum time weighted average exposure value ascribed by the United States National Institute of Occupational Safety and Health is 35 ppm over an 8 h period [44]. The gas is mainly a product of poorly combusted organic material, such as petrol, oil or gas. Carbon monoxide is constantly in the public eye, largely because the home is such a susceptible place for carbon monoxide poisoning; usually from a faulty gas powered boilers that lead to annual deaths. Carbon monoxide concentrations are particularly high in areas of industry, where fossil fuels are combusted for energy purposes, and in cities where there are high levels of traffic. Existing sensors are used in homes as a warning system to the otherwise undetectable carbon monoxide. Sensors fall in to two main types; ‘blob’ sensors, and electrical sensors: ‘Blob’ sensors are essentially a patch of metal oxide salts that upon interaction with the monoxide reduce the salt, forming carbon dioxide. The salt turns black when it is reduced, the colour change being the feature alerting the observer. Though cheap, the alerting system requires the vigilance of the observer in order to recognise the change in concentration. Given that at high concentration carbon monoxide causes dizziness and confusion, the occupant may be in no condition to readily observe this change. Electronic carbon monoxide sensors come in two main types: thermistor type metal-oxide detectors; detecting a change in heat when carbon monoxide lands on the oxide and reacts, (the change in temperature raising the alarm), and an electrolytic detector that works by sensing the change in charge carriers in an electrolyte solution when carbon monoxide interacts with an electrode of the device.

Carbon monoxide gas sensors have a myriad of applications, not just for home safety, also in measuring atmospheric concentrations, in the exhaust of cars, and for process monitoring in industrial plants.

### 2.2. Advances in Carbon Monoxide Sensors

**Figure 7.**
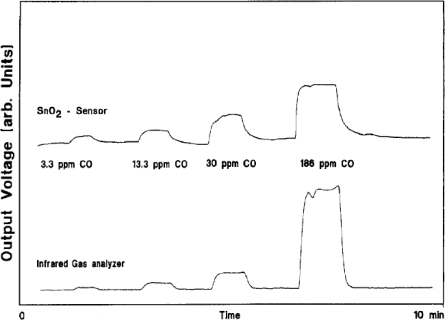
Showing the sensor response to varying concentrations of carbon monoxide for both a SnO_2_ sensor and an IR gas analyser. Figure adapted with permission from [45].

Sensors have been utilised by researchers to actively measure the varying concentration of CO in the environment. Wiegleb and Heitbaum [45] reported the use of metal oxide gas sensors as detectors for monitoring NO and CO gas concentrations in cars, studying the variation in concentration over an extended period. SnO_2_ was used to detect the change in concentration of CO and In_2_O to detect the concentration of NO. The sensor was used to measure the concentration of monoxide at varying stages of the journey; metal oxides were used due to their fast response to the change in concentration. These responses were compared with an infrared gas analyser (BINOS 1, Leybold-Heraeus GmbH) (Figure 7). The responses are comparable although the IR analyser has a greater dynamic range the MOS device had a greater response at low concentrations.

Barbi *et al.* [46] developed SnO_2_ based sensor that showed a response to the presence of CO gas from concentrations of 10 ppm and above. The ideal operating temperature was shown to be 250 °C, with the response (R_0_/R) showing 2.2 at 20 ppm, and 4.1 at 100 ppm, giving a steady increase with concentration.

**Figure 8.**
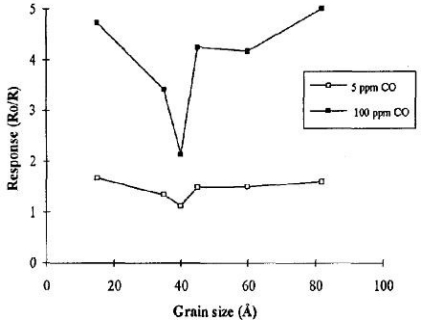
The effect of grain size on the size of response to the carbon monoxide target gas. Figure adapted with permission from [46].

Doping the material with platinum gave a change in the structure, and in general the platinum decreased the sensitivity and a slower response time compared to the pure tin oxide material. The authors [30] also demonstrated the advantages of control over grain size (Figure 8). The largest responses to the gas were found at grain sizes of 20 angstroms, a result of the whole grain behaving like a surface and at 80 angstroms, attributed to areas between grains that retain charge in the structure as seen in the equivalent circuit model.

Riviere *et al.* [47] used screen printing to produce a tin oxide film, varying the composition of the precursor ink. The materials demonstrated a strong response to the presence of carbon monoxide, showing an increase of approximately 1 log Ohm^−1^ CO at 300 ppm, and a temperature of 500 °C, though it should be noted that the CO was only introduced after an hour in the presence of air, as the conductance had to be at an appropriate level.

**Figure 9.**
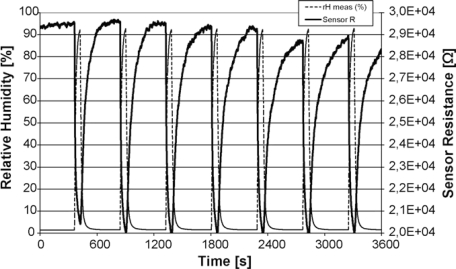
Demonstrating the response of the sensor to humidity. Note the decrease in baseline response over time and successive introductions. Figure adapted with permission from [30].

Tischner, in 2008 [30] announced the development of a SnO_2_ thin film (50–100 nm) deposited by a spray pyrolysis method. The films were found to operate best in the region of 250–400 °C, and sensed CO concentrations at concentrations of 5 ppm, concentrations expected in a normal home. When tested, the film showed a temperature dependence on the response: At 250 °C, the film showed good responses between 0–40 ppm, at 400 °C, the film increased its response range to between 0 and 100 ppm. The film was also found to be extremely sensitive to humidity (Figure 9), a problem for applications in the field, where variable humidity is expected. The authors suggest thinner films might be more sensitive, due to the grain boundary effects mentioned in the introduction (Figure 2).

**Figure 10.**
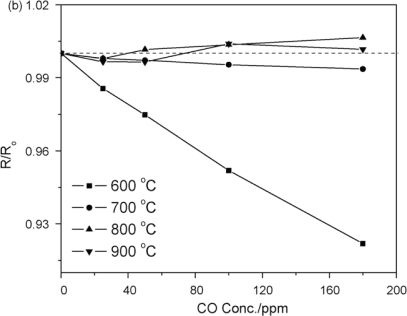
The response of rutile film to carbon monoxide at various temperatures. Figure adapted with permission from [48].

Li *et al.* [48] compared different phases of titanium oxide (TiO_2_) films. Anatase TiO_2_ was found to show an n type response to the presence of CO. The rutile film exhibits n and p type responses depending on the temperature (Figure 10) at which the readings were taken, and the concentrations of both oxygen, and carbon monoxide. This demonstrates that the phase of the material produced when making a metal oxide semiconductor sensor is important, and must be taken in to account when choosing the deposition method and conditions.

Izu *et al.* [49] studied the response of cerium oxide films after they had been sintered at varying temperatures (between 800 and 1,030 °C). The film formed after sintering at 950 °C showed the best response to the presence of CO at a temperature of 450 °C, and a concentration of CO at 5,000 ppm. Other cerium oxide films have also been developed using electron beam evaporation, and then annealing at 500 °C (Durrani *et al*. [50]). The optimum operating temperature of the films was found to be 390 °C, with the best response showing when CO was at a concentration of 500 ppm. The response times were slow with this material however, taking 45 s to respond to the CO, and 25 s for the film to recover in the absence of the target gas.

### 2.3. Outlook for MOS Carbon Monoxide Sensors

As things currently stand the best metal oxide semiconductor material for the detection of carbon monoxide is SnO_2_. Others, such as CeO_2_ and TiO_2_, have been investigated but are yet to match the performance of SnO_2_ systems having poor responses at low CO concentrations. Such sensors have been demonstrated to have higher responses to carbon monoxide at low concentrations than an infrared gas analyser [51] and as a result show great promise for cheaper air quality measurement. The use of operating temperatures around 250 °C helps to lower power requirements, although it is still desirable to minimise these further, as this remains the single largest stumbling block to the use of MOS technology. The use of small particles sizes, around 2 nm, has been shown to increase gas response, although material processing in device fabrication has yet to be optimised in this instance. The investigation of nanomaterials provides a potential way forward for future research into lowering device power consumption and increasing gas response.

## 3. Carbon Dioxide Sensors

### 3.1. Reasons for Detecting Carbon Dioxide

Carbon dioxide is present in air at concentrations (as of January 2010) of 388 ppm in the Earth’s atmosphere [52]. Carbon dioxide has many uses: carbonating drinks, pneumatic applications, fire extinguishers, photosynthesis; it is an essential ingredient for the process to take place, lasers and refrigerants are just some examples where carbon dioxide has found a use, by nature and by humans.

**Figure 11.**
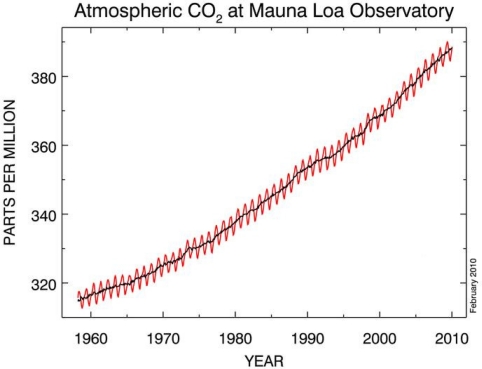
A graph depicting the steady increase in CO_2_ concentrations since 1958 at the Mauna Loa Observatory. Figure adapted from [52].

Carbon dioxide is also a greenhouse gas. In the atmosphere, it absorbs infra-red energy, and vibrates, a process which then passes heat to its surroundings, increasing the ambient temperature. If the concentration of carbon dioxide increases in the atmosphere, the temperature increases too. Thus, accurate, reliable sensors with an appropriate temperature range could be used to measure the change in concentration. The recent increase in the concentration of carbon dioxide through human activity (the combustion of hydrocarbons and other carbon containing fuels such as coal, methane, petrol and kerosene with an appropriate amount of oxygen yields carbon dioxide and water) is leading to concern about whether humans are causing global warming. Sensors can measure the output of CO_2_ during combustion, thus giving important real-time information as to the amount of carbon dioxide being produced by the activity. Atmospheric carbon dioxide concentrations have risen steadily since 1958, and are expected to carry on rising as long as man satisfies its thirst for combusting fossil fuels (Figure 11).

Carbon dioxide can cause substantial negative health affects to humans (Table 3) including drowsiness and at high enough concentrations suffocation.

The maximum time averaged exposure recommended by the United States Occupational Safety and Health Administration is 5,000 ppm over eight hours [53]. As such it is highly desirable to be able to measure carbon dioxide in order to control indoor air quality.

### 3.2. Advances in Carbon Dioxide Sensors

Patel *et al.* [55] fabricated a indium tin oxide (ITO) sensor demonstrating a sensitivity to carbon dioxide. The 150 nm films were made by thermal evaporation of precursors containing tin and indium. The material was annealed in humid air, at 700 K for 1 h. The response to carbon dioxide was tested while varying the temperature. The responsivity was proportional to the working temperature and the concentration of the target gas. Concentrations of carbon dioxide tested were 200 ppm, 500 ppm and 1,000 ppm, with the ideal working temperature found to be at 573 K.

**Figure 12.**
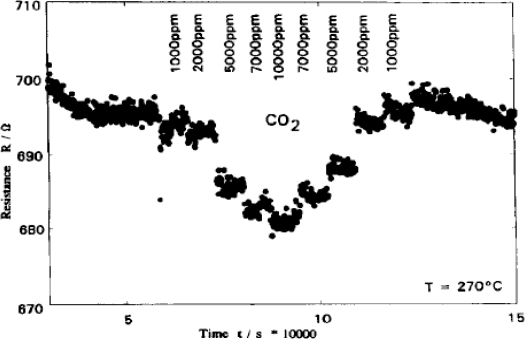
response of the SnO_2_ film developed by Hoefer *et al.* to CO_2_ gas in synthetic air at 270 °C. Figure adapted with permission from [56].

Hoefer *et al.* [56] reported the development of a sensor film based on SnO_2_. The sensor showed a marked response at a concentration range of 1,000–10,000 ppm range at 270 °C. The sensor material starts showing significant responses above CO_2_ concentrations of 5,000 ppm: between 2,000 and 5,000 ppm the material is most responsive. Between 5,000 and 10,000 ppm there is only a small change in response (1–2 ohms); and likewise between 1,000–2,000 ppm the sensors show a similar change. The sensor is responsive across a large range of CO_2_ concentrations (Figure 12), but can only effectively differentiate between large concentrations (thousands of parts per million), and as such has only limited application.

**Figure 13.**
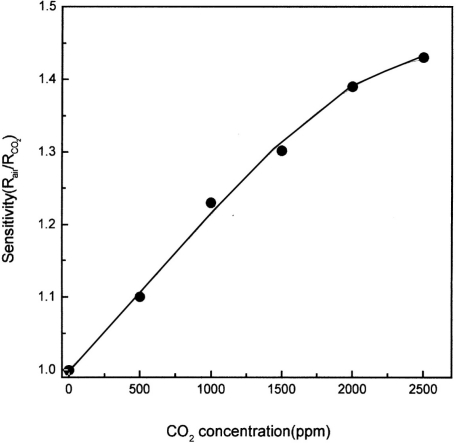
The responsivity change of the Lanthanum film to carbon dioxide upon increasing concentration (temperature unreported). Figure adapted with permissions from [57].

Kim *et al.* [57] studied the precursor LaCl_3_·7H_2_O by heating the material at a range of temperatures (400–1,200 °C), creating different compositions. Kim found that the material adopted a composition of LaOCl when heated to 400 °C, at 800 °C the La_2_O_3_ composition was created. The material was heated in this range, and thus deposited on top of a SnO_2_ thick film. The sensitivity of the film was investigated, against the temperature at which the film was treated. Heat treatment at 400 °C yielded little or no response to the target gas, but at 800 °C there was a large change in the response, with a value of 1.3, heating at 1,000 °C was found to give the film with the highest sensitivity to carbon dioxide (a response value of 1.37). The responsivity showed a linear trend between responses (R/R_0_) at concentrations of 0–2,500 ppm (Figure 13). The sensor developed demonstrates the response properties of LaOCl, though investigations as to responses to other gases have not been made. It was found that the sensor film needed to be heated to 1,000 °C in order to be effective; such high temperatures may not be compatible with some sensor substrates.

**Figure 14.**
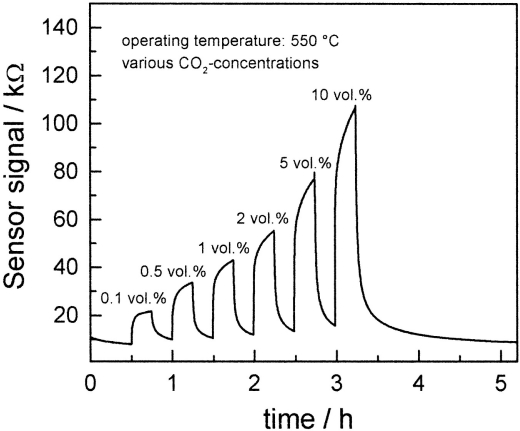
The effect of varying CO_2_ concentration on the signal of the BaTiO_3_ sensing material (note the baseline increase over time). Figure adapted with permission from [58].

Lee *et al.* [58] produced microcrystalline films with thicknesses of 2–5 μm made from doped BaTiO_3_ that showed extremely good sensitivity to the presence of CO_2_. A paste was screen printed on to a sensor surface, dried at 80 °C, then heated at 750 °C for 23 minutes. After testing a range of doped materials in air with 1% CO_2_ content, the most sensitive material was found to be BaTiO_3_-LaCl_3_ (10% wt). The sensors signal was found to increase with CO_2_ concentration at 550 °C (20 kΩ for 0.1% vol, 32 kΩ for 0.2% vol to 110 kΩ for 10% vol). Along with the sensitivity to concentration, the sensor demonstrated a rapid response to the presence of the target gas at 550 °C (Figure 14).

**Figure 15.**
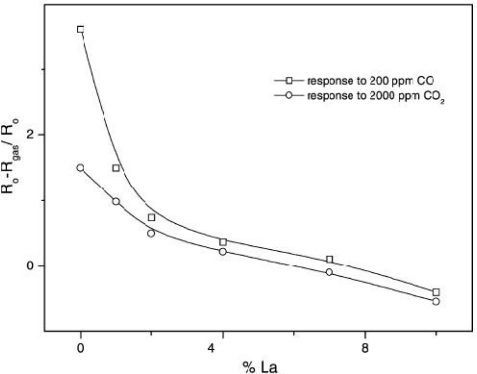
Results demonstrating the effect in changing La concentration on the responsivity of the SnO_2_ film, also showing poor discrimination between carbon monoxide and carbon dioxide. Figure adapted with permission from [59].

Marsal *et al.* [59] demonstrated the response of La (under varying amounts) doped tin oxide (SnO_2_) films after they were annealed at 450 °C under N_2_ for 25 min. The un-doped tin oxide showed a good response to carbon dioxide at 100 °C, other doped materials showed better responses, but at higher operating temperatures (400 °C). The largest response of the doped materials was the tin doped with BaTiO_3_-CuO-La_2_O_3_, which responded at 550 °C. The percentage of lanthanum in the films was found to be critical in optimising the gas response (Figure 15).

Marsal *et al.* [60] also reported a sensor based on LaOCl that showed an ability to detect CO_2_. screen-printing produced a film that showed a response of 3.4 to 2,000 ppm CO_2_. The sensor gave this optimum response at a temperature of 260 °C. The authors also tested the response to CO, this screen printed sensor was far more responsive to CO_2_ than CO (a response difference of around 2.5–3). The authors conclude by saying the sensitivity of the material and the lower operating temperatures needed for the material to function make the LaOCl material the best yet produced for detecting CO_2_.

### 3.3. Outlook for MOS Carbon Dioxide Sensors

A number of different metal oxide semiconductor materials have been investigated for the detection of carbon dioxide including ITO, SnO_2_, BaTiO_3_ and LaOCl. Of these LaOCl has the best performance. CO_2_ detection is problematic because the standard theory of surface interaction affecting the band structure and charge carrier concentration of the material appears to be insufficient as a guide to improving the gas response. As such the working range reported in the literature typically covers the range 2,000–10,000 ppm. Developing the ability to reliably measure concentrations in the 500–2,000 ppm is the biggest challenge facing MOS sensors for carbon dioxide detection at the current time. Given that traditional approaches such as doping seem to be ineffective in improving sensor performance; new materials need to be developed in order to meet this challenge and more work understanding the sensing mechanism for CO_2_ needs to be conducted to aid materials discovery. The development of LaOCl goes someway to meeting this challenge but ultimately there is still a long way to go.

## 4. Nitrogen Oxide (NO_x_) Sensors

### 4.1. Reasons for Detecting Nitrous Oxides

NOx are a varied group of gases, the simplest form being nitric oxide; NO, then nitrogen dioxide; NO_2_, nitrous oxide; N_2_O, dinitrogen trioxide; N_2_O_3_, dinitrogen tetroxide; N_2_O_4_ and dinitrogen pentoxide; N_2_O_5_. The gases are principally formed from the combustion of fossil fuels in internal combustion engines, where the energy of the combustion reaction helps combine nitrogen gas (N_2_) and oxygen gas (O_2_). Nitric oxide is a known component of photochemical smog, combining with hydrocarbons and oxygen to form a thick cloud over heavily industrialised areas. Photochemical smog, apart from the haze it produces, is irritating to the eyes, and also damages plant life in the affected areas. The monitoring of the concentrations of NO in the air can be particularly useful, as environmental agencies can use it to predict how likely smog is to form, thus being able to alert the public at times of significant risk.

Nitrogen dioxide is toxic upon inhalation, but unlike carbon monoxide, it is easily detected by smell, one complicating factor of this is that exposure to the gas at 4 ppm anaesthetises the nose creating the possibility that increased concentrations in an environment may go unnoticed, causing potential health risks. The main risks of nitrogen dioxide appear to be their effect on the lungs. People with bronchitis or asthma are particularly sensitive to the gas, and lungs may become inflamed, leading to breathing difficulties. Animal subjects subjected to long-term exposure of NO_2_ were found to have damaged lungs [61,62]. Studies have linked the concentration of NO_2_ and SO_2_ to the exacerbation of conditions such as chronic bronchitis and emphysema [63]. The monitoring of the gas in environments where NO_2_ is at particularly high risk concentrations is desirable as one would like to reduce the risks to human health as much as possible.

### 4.2. Advances in Nitrous Oxide sensors

Akiyama *et al.* [64] produced a sintered film of WO_3_ which was found to be very sensitive to both NO and NO_2_ gases. The authors found that the film was most responsive to the presence of 200 ppm NO gas at 200 °C. When sensing NO_2_ gas, the sensor showed a large response at 300 °C, with the sensor dropping its response back down to pre-gas introduction levels at a very fast rate. When testing the material in the presence of other gases such as CO, hydrogen and methane, the sensor showed an extremely low response, showing that these sensors were able to discriminate between gases.

**Figure 16.**
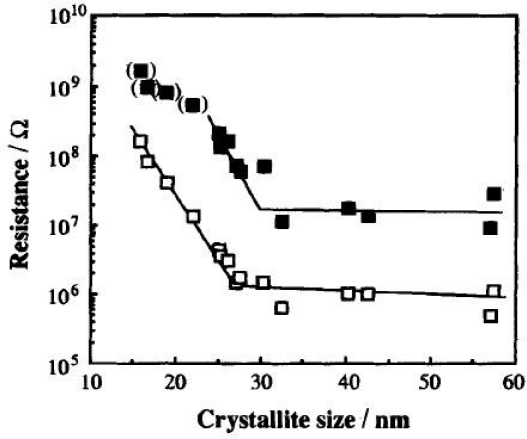
The variation in resistance according to WO_3_ crystallite size for; □ air, and ▪5 ppm NO_2_ in air at 300 °C. Figure adapted with permission from [65].

Tamaki *et al.* [65] observed the effects of grain size of WO_3_ on the response of a WO_3_ sensor. The crystallite sizes ranged between 16 and 57 nm. In varying the size of the grains the response of the material to the target gases varied (Figure 16). The grains were most sensitive to both NO and NO_2_ at sizes of 25 nm, the sensitivity increasing with concentration of the target gas.

Cantalini *et al.* [66] produced films of WO_3_ of 150 nm thickness. The films were deposited, and then annealed at temperatures of 400, 500 and 600 °C for 1 h. The largest responses to NO_2_ gas were found at working temperatures of 200 °C using the film annealed at 500 °C. Varying the concentration of the NO_2_ gas at 350 °C demonstrated sensitivity to concentrations between 0.2 and 5.0 ppm. When testing the responses of the materials in the presence of other gases, the presence of CO and methane showed no change in response, however, the presence of ethanol and water vapour did alter the response. The effect of annealing temperatures is shown to have an effect on the properties of the film, and matching these temperatures to the requirements of the film is an important consideration.

**Figure 17.**
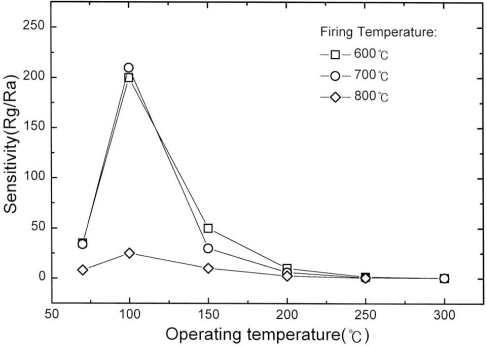
Demonstrating the effect of firing temperature on the responsivity of the WO_3_ film. Figure adapted with permission from [67].

Chung *et al.* [67] made thick-film sensors made of WO_3_ using a screen printing method, the paste having been formed from ball milling WO_3_ powder with ethanol for a day. The sensors were dried and fired for 1 h under 600, 700 and 800 °C, achieving films of about 30 μm. Films performing under the operating temperature of 100 °C, having been fired at 700 °C showed fast response times, good sensitivity and good recovery times. At 800 °C, the resistance of the film increases, interaction with the target gas no longer has an effect on the conductivity of the film; this can be attributed to a change of phase in the material due to the increase in deposition temperature (Figure 17). Heat treatment in 40–50 % oxygen atmospheres gave the highest sensitivity, control of oxygen content in the film was important in sensitivity control for WO_3_. The low operating temperatures and high sensitivity of the material produced in this paper supports screen-printing as an effective deposition method for WO_3_ film formation.

Lee *et al.* [68] investigated the effects of adding TiO_2_ in to a WO_3_ film. Three films were investigated: the un-doped WO_3_ film, a film of WO_3_ doped with 4% TiO_2_; denoted as TW and a film of (WTi)O_3_; denoted as NTW. The responses of the material to the presence of NO_2_ varied according to film type. NTW showed the largest sensitivity to NO_2_, exhibiting a response at 175 °C, with the sensitivity peaking at 225 °C. TW showed a lower sensitivity at all temperature ranges, peaking at 270 °C. The un-doped WO_3_ film exhibited very low sensitivity comparatively, and showed little temperature dependence. The sensor could be used to monitor NO_2_ gas levels in exhausts at ppm concentrations.

Comini *et al.* [69] showed a film formed by magnetron sputtering of tin oxide to be sensitive to nitrogen dioxide. The deposition occurred on a silicon wafer, in an inert argon atmosphere, at low pressures (0.007 mbar), the films had a high porosity. The films were annealed, but the temperature is not reported. Upon the introduction of NO_2_, the sensor showed a significant decrease in current in response to the gas. The responses are obvious at just 1ppm and are large at 5 ppm and 10 ppm. The investigations in to the response were carried out at 300 °C. The film does show a response time of 2–6 mins, a relatively slow response time for metal oxide semiconductor gas sensing film.

**Figure 18.**
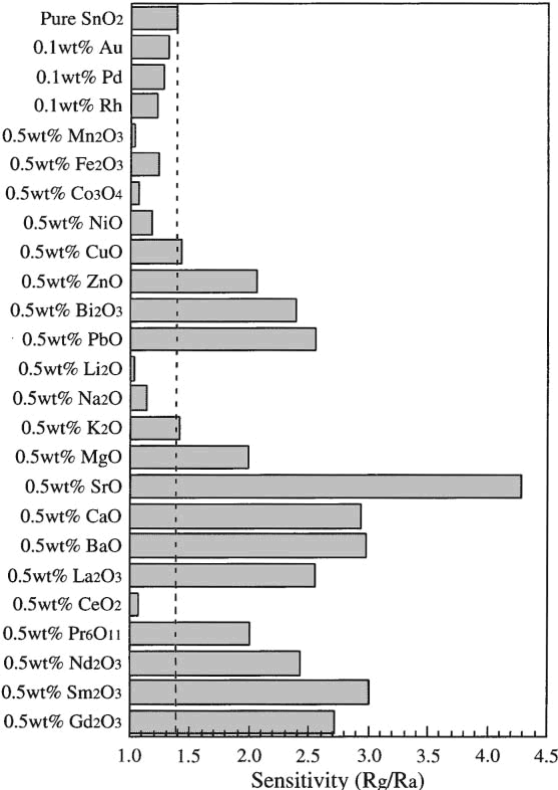
The effect of doping the material with various oxides on the responsivity of SnO_2_ to N_2_O gas. Figure adapted with permission from [70].

Kanazawa *et al.* [70] used a large range of metal oxide semiconductor sensors to investigate the detection of N_2_O for medical purposes (Figure 18). The authors first tested the varying pure oxides on their own, most oxides showing little or no response. The best responses were found with SnO_2_, WO_3_, and ZnO all at a temperature of 450 °C. The authors then doped SnO_2_, the best material with varying metal oxides, and found that the material doped with 0.5 (by mass) SrO gave a material with an increased response of 4.25, compared to the original 1.66 (both calculated.using R/R_0_).

Shieh *et al.* [71] produced WTiO and WO_3_ films using sol-gel dip coating. The sensing films were on an alumina substrate, after four coatings, the samples were calcined at 400 °C for 1 h. The sensitivities of the two films were compared. WO_3_ at 10 ppm NO_2_, with a temperature at 200 °C showed a sensitivity of 82%, compared to a sensitivity of 1,786% for the WTiO film. The large difference in sensitivity is attributed to the reduction in grain size of the material and a change in the debye length of the grain. Changing the physical properties of the film by doping with a metal such as Titanium shows an effective approach to increasing sensitivity.

Tomchenko *et al*. [72] investigated the use of several metal oxides working together with a view to monitoring the concentrations of certain gases in an environment, in particular an exhaust. SnO, ZnO, WO_3_, In_2_O_3_ and CuO were investigated. Materials demonstrated different responses to NO and NO_2_, and also had markedly different responses at different temperatures. At 200 °C, the In_2_O_3_ showed the highest response, with CuO showing no response at all, and ZnO showing a very poor response. At 400 °C, the response changes, ZnO shows the largest response and CuO still demonstrating no response to the presence of the gas. The response times for 200 °C were about 10–30 s. The researchers argue that identifying the responses could lead to the incorporation of materials to monitor gases in a number of environments, possibly in an electronic nose.

**Figure 19.**
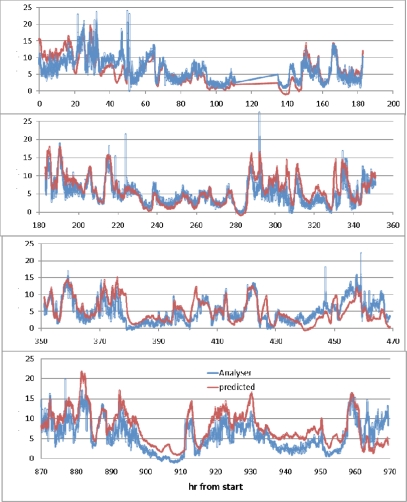
Graph comparing the responses of chemiluminescence and semiconductor sensors. Measurements were simultaneous and made every minute. X-axis indicates hours since experiment start and y-axis ppb concentration of NO_2_. Figure adapted with permission from [73].

The development of a sensor for ozone (O_3_) and nitrogen dioxide (NO_2_) with sensitivities at less that 10 ppb was reported by Williams *et al* [73]. The sensor is based on a film of tungsten oxide (WO_3_) of ∼90 μm thickness, screen-printed on to gold electrodes. The authors reported that the sensitivity is strongly related to the operating temperature of the sensor, and the temperature was very closely monitored and controlled. Though the response times were short for the film, the sensor exhibited a linear response between gas concentration and resistance, with an error of ±2 ppb, the reference detector being a chemiluminescence sensor instrument. The responsivity of the tungsten sensor was tracked with the reference sensor over monthly periods, showing no change in response and matching the readings from the reference sensor. Williams then compared the results between the chemiluminescence sensor and the tungsten sensor, finding the sensors to be virtually equal in response. The equality suggests that metal oxide sensors could be preferred, as the sensors are far cheaper than their chemiluminescence analogues with similar response patterns (Figure 19).

### 4.3. Outlook for MOS Nitrous Oxide Sensors

WO_3_ is the most widely used material for the detection of nitrous oxides—typically NO_2_ and NO. It provides the largest response to NO_2_ of all MOS materials with minimal cross sensitivity (except in the case of ozone) and it finds use in many commercial products. New approaches have looked to improve the performance of WO_3_ based devices further; tailoring grain size and film thickness have both shown improvements in gas response whilst careful control of oxygen content and operating temperature have helped to optimise material sensitivity. Currently WO_3_ MOS sensors are capable of measuring accurately in the 10 ppb range, well within the safe limit for human exposure.

Other nitrous oxides have had less attention devoted to them, understandably as these gases are less common and of less concern than NO_2_ and NO, although promising work has been conducted on the detection of N_2_O where doping studies have clearly shown that doping SnO_2_ with 0.5 wt.% SrO leads to a large enhancement in response although no information on cross sensitivity is reported for this system. The further use of mixed metal oxides shows promise for the development of nitrous oxide sensors.

## 5. Ammonia Sensors

### 5.1. Reasons for Detecting Ammonia

Ammonia, NH_3_ is a caustic and hazardous colourless gas with a characteristic pungent odour. The worldwide production of ammonia in 2007 was estimated at almost 140 million tonnes, a rise from approximately 2 million tonnes in 1945 [74]. 83% of the world’s ammonia was used in the fertilization of crops in 2003 (global production was around 115 million tonnes) [74]. Ammonia is a source of nitrogen to living organisms, nitrogen being present in vital biological molecules such as amino acids (the building blocks of proteins).

Ammonia is present in animal waste, and in particularly high concentrations in chicken faeces. In agricultural environments, where animals can live in unventilated environments, ammonia concentrations can become high enough to kill the animals. An ability to monitor and control these environments is highly desirable.

### 5.2. Advances in Ammonia Sensors

Nanto [75] reported a zinc oxide thin film sensor prepared by sputtering. The material was doped with aluminium, gallium and indium; the response of these materials was compared with the un-doped film. Large negative responses are reported in reaction to the presence of ammonia, with positive responses to other gases depending on the material (Table 4).

The large negative response of the material to the ammonia gas, coupled with positive responses in the presence of other gases is encouraging. The authors do not test effect of water vapour on the response of the material, one of the most important disruptors of response. Though the large negative readings are good, the effect of the other gases on the sensor response indicates this sensor is highly cross sensitive and therefore has limited use.

Wang *et al.* [76] investigated the effects of incorporating 1% weight amounts of varying metal oxides in to the WO_3_ structure, and the resulting response to the presence of NO and NH_3_ gas were investigated. The preparation method involved calcining a pre-prepared WO_3_ powder on a sensor fitted with Pt electrodes for 1 hour at 600 °C. The response testing compared 30 ppm NH_3_ and 40 ppm NO. The most effective dopants were found to be magnesium, zinc, molybdenum and rhenium; molybdenum giving the highest reading. The authors also demonstrated that some dopants made the material specific to one gas or the other. Materials doped with chromium, lanthanum, promethium, samarium, gadolinium, europium, thulium and ytterbium showed little or no response to the presence of NO but responses to NH_3_.

TiO_2_ films on silicon prepared by magnetron sputtering were shown by Karunagaran *et al.* [77] to increase their conductance in the presence of ammonia gas. The initially deposited film showed no response to the target gas. The authors annealed the film at 600 °C and the films then showed a sensitivity range at 500–1,000 ppm (this was the testing range at the films optimum temperature, 250 °C, there is no data outside this range). A response time of 90 s was reported.

**Figure 20.**
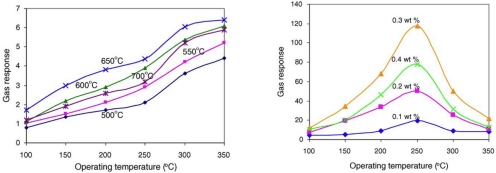
Left, The responses (as %) of pure ZnO thick films fired at varying temperatures to 1,000 ppm of ammonia. Right, showing the optimum operating temperature for ZnO films for sensing 1,000 ppm ammonia with RuO_2_ doping. Figure adapted with permission from [78].

Wagh *et al*. [78] used screen-printing to make pure and RuO_2_-doped ZnO thick films (Figure 20), the thickness of the films was not reported. The materials were fired between 500 and 700 °C at 50 degree intervals, the most sensitive films were fired at 650 °C. The un-doped films showed little response to the target gas. All the doped films tested showed good responses at operating temperatures between 100 and 350 °C, the higher the operating temperature, the better the response to the gas, though the low temperature response at 100 °C is promising. The optimum doping amount was seen to be 0.3% wt, with the doped materials showing the largest response at 250 °C.

### 5.3. Outlook for MOS Ammonia Sensors

A variety of materials have been investigated for the detection of ammonia gas including ZnO, WO_3_ and TiO_2_. None of the materials are clearly ahead of the others in terms of performance. Doping studies have been conducted with some success in improving gas response and lowering operating temperature. Future studies on the effects of grain size ought to take place to improve sensor response further and in the case of WO_3_ based sensors doping studies expanded to improve selectivity toward ammonia and limit cross sensitivity to NO and NO_2_.

## 6. Recent Work Conducted at University College London

### 6.1. Zeolite Modified Sensors

**Figure 21.**
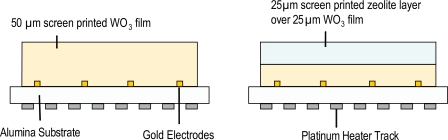
Schematic of sensor fabrication, Left a standard screen-printed sensor, Right a sensor modified with a zeolites over-layer.

Zeolites are porous aluminosilicate structures that are able to accommodate cations such as Na^+^, Mg^+^, Mg^2+^ and small molecules such as ethanol *etc*. These cations / molecules are contained within a large 3-dimensional framework, with external pore sizes typically ranging from 4–12 Å. The oxygen atoms in the zeolites structure may be negatively charged due to aluminium substitution of silicon; this charge can be used to attract other species, commonly a cation such as Na^+^ or H^+^. As a result zeolites may behave as selective catalysts and are capable of discriminating between gas molecules on the basis of size and shape, allowing some gases through their structure, whilst not admitting others. A schematic of sensor fabrication is given in Figure 21.

Further to this the zeolites can behave in a chromatographic manner through variable diffusion characteristics. A mixture of gas may enter the zeolites pores, but different binding strengths to the zeolite interior leads to a difference in diffusion speed through the zeolites. Finally the zeolites may perform a catalytic reaction involving the target gas, this may lead to the production of one or more molecules that the sensor element may be more or less sensitive too. In the ideal case, this catalytic reaction will be specific to a particular analyte and lead to the production of multiple species that the sensor element is more sensitive too, leading to a large enhancement in response signal for a given analyte with no chance of cross sensitivity. Limited amounts of work have been conducted on the use of zeolites in this fashion [79–82] indicating a differential response as a result of zeolite layer incorporation.

**Figure 22.**
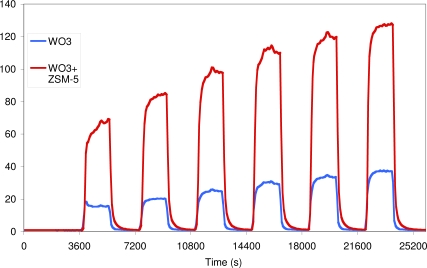
Gas responses of tungsten oxide and zeolites modified tungsten oxide sensors to 100, 120, 140, 160, 180, 200 ppb NO_2_ in dry air at 350 °C.

Recent work conducted at UCL [12,83–85] suggests that it is possible to utilise zeolites as transformation layers to improve both the gas response and selectivity of metal oxide semiconductor gas sensors. Figure 22, gives an example gas response to nitrogen dioxide for a standard screen-printed tungsten oxide sensor and a sensor that has been modified with an over-layer of H-zeolite B. As can be seen from the figure the modified sensor gives a considerably larger gas response than the unmodified sensor due to a catalytic reaction taking place within the zeolites pores. The sensor also proves to be more selective as the zeolite layer also acts as a size and shape selective filter for the sensing element, essentially excluding potential interferents such as larger hydrocarbon molecules, branched alcohols *etc*., from reaching the sensor element and disrupting the nitrogen dioxide response.

### 6.2. Carbon Dioxide Sensors

**Figure 23.**
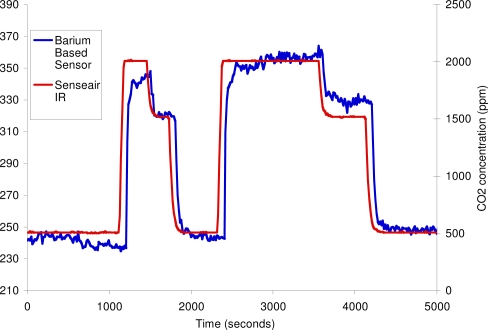
Comparison of CO_2_ Sensor Technologies: Commercial Senseair Infra-red device *versus* a barium based metal oxide semiconductor sensor developed at UCL. CO_2_ concentration indicated by the K30 is plotted on right hand axis and the UCL sensor response is on left hand axis, showing a change in resistance in the MOhm region.

Perovskite compounds such as BaTiO_3_ and BaSnO_3_ [58] have been investigated for their capacitive gas response to carbon dioxide. These sensors had drawbacks as they experience a large cross sensitivity to water. In our labs at UCL we have developed a new barium based material by traditional ceramic powder processing methods and the devices are prepared using conventional screen-printing techniques. Sensors made with this material gave a significant chemiresistive response to carbon dioxide with minimal signal disruption from humidity that is comparable to the response of a commercial optical sensor from Senseair (model number: K30) (Figure 23).

### 6.3. Electric Fields and Chemical Vapour Deposition (ElFi CVD)

**Figure 24.**
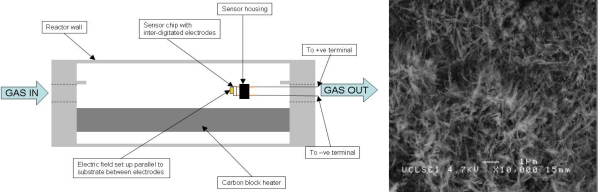
(Left) Schematic illustrates the CVD reactor design used in the production of sensors using ElFi CVD. (Right) Example morphology of vanadium dioxide thin films grown on sensor substrates from the ElFi CVD reaction of vanadyl acetylacetonate at 525 °C with an applied AC electric field.

The gas sensor substrates to be used have inter-digitated electrodes, which allow CVD reactions with an electric field parallel to the substrate to be set up. The electrode array comprises of gold electrode “fingers” 100 microns wide and 10 microns thick. Typically the electrode gap is 40 microns though a variety of substrates with different electrode gaps have been used (40, 80 and 150 microns). The sensor substrates can easily be incorporated into pre-existing deposition equipment (Figure 24 left). This technique has led to the production of fibrous growth and high surface area morphologies (Figure 24 right). This indicates the suitability of this method for the preparation of next generation metal oxide semiconductor devices.

## 7. Conclusions

The development of semiconducting metal oxides as gas sensors has accelerated over the past 20 years. Increasingly (as we have briefly shown) these sensors are being used increasingly in the monitoring of environmentally important gases. Metal oxide semiconductor sensors have been shown to be sensitive to a large range of gases, with responses varying with target gas concentration and device operating temperature. These properties can be tailored the specific environment in which the sensor is to be used, by understanding the material science involved. Advances have been made in the understanding of materials chemistry and materials processing such as doping, deposition temperatures, and annealing temperatures. These have been shown to have a profound effect on the material structure and subsequently the gas sensing properties of the sensors. Modification of the surface by selective and catalytic materials such as zeolites is an important development, and shows a further way in which the materials’ science can be used to further improve the specificity and response to a target gas, whilst making sure the response of the film is not disrupted by a competing gas, or humidity. New materials and techniques continue to be developed to push the abilities and properties of gas sensors. Currently metal oxide semiconductor sensors find limited commercial use; other types of sensor are still favoured for many applications. However, with recent advances in understanding of materials chemistry and synthetic techniques, their properties, coupled with their relative low cost could lead to them becoming ever more important tools in environmental monitoring.

## Figures and Tables

**Table 1.** Sign of resistance change (increase or decrease) to change in gas atmosphere [6].

**Classification**	**Oxidising Gases**	**Reducing Gases**
n-type	Resistance increase	Resistance decrease
p-type	Resistance decrease	Resistance increase

**Table 2.** Demonstrating the effects of certain concentrations of carbon monoxide on human health. COHb is the percentage of carbon monoxide in the blood. Typical concentrations in the home are 0.5–5 ppm (united states environmental protection agency).

**Carbon Monoxide Concentration (ppm)**	**COHb Level (%)**	**Signs and Symptoms**
35	<10	Headache and dizziness within 6 to 8 hours of exposure
100	>10	Slight headache in 2 to 3 hours
200	20	Slight headache within 2 to 3 hours; loss of judgement
400	25	Frontal headache within 1 to 2 hours
800	30	Dizziness, nausea and convulsions within 45 minutes, insensible within 2 hours
1,600	40	Headache, tachycardia, dizziness and nausea within 20 minutes; death within 30 minutes
3,200	50	Headache dizziness and nausea in 5 to 10 minutes; death within 30 minutes
6,400	60	Headache and dizziness in 1 to 2 minutes; convulsions, respiratory arrest, and death in less than 20 minutes
12,800	>70	Death in less than 3 minutes

**Table 3.** Detailing the effects of concentration and exposure time of CO_2_ on human health Taken from occupational health and safety website in Canada [54].

**CO_2_ Concentration and Exposure time**	**Effect on Health (symptoms)**
0.035%	Approximate atmospheric concentration, no noticeable effect.
3.3–5.4% for 15 mins	Increased depth of breathing
7.5% for 15 mins	Feeling of an inability to breathe, increased pulse rate, headache, dizziness, sweating, restlessness, disorientation, and visual distortion.
3%, for over 15 hours	Decreased night vision, colour sensitivity
10%, 1.5 mins	Eye flickering, increased muscle activity, twitching
10+%	Difficulty in breathing, impaired hearing, nausea, vomiting, a strangling sensation, sweating, after 15 minutes a loss of consciousness.
30%	Unconsciousness, convulsions. Several deaths attributed to CO_2_ at concentrations of more than 20%

**Table 4.** Showing the resistance change of different forms of ZnO (doped and un-doped) in the presence of different gases. Taken from H. Nanto [75].

**Gas Species**	**ΔR for ZnO**	**ΔR Al-doped ZnO (2% wt)**	**ΔR In-doped ZnO (2% wt)**	**ΔR Ga-doped ZnO (2% wt)**
Ammonia	−72	−94	−88	−82
Hydrogen	35	13	15	49
Butane	21	0	0	2
Methane	0	0	0	0
Ethyl Alcohol	69	54	39	40
Acetone	89	84	62	68
